# Standardizing wrist x-rays: The human-centered design and evaluation of a wrist x-ray positioning tool

**DOI:** 10.1371/journal.pone.0359437

**Published:** 2026-10-05

**Authors:** Laura Vancer, Maxwell Campbell, Glenn Schurmans, John Henry, Jamie Kueneman, Joy MacDermid, Gregory Garvin, Emily Lalone

**Affiliations:** 1 School of Biomedical Engineering, Western University, London, Ontario, Canada; 2 Roth McFarlane Hand and Upper Limb Centre, St. Joseph’s Health Care, London, Ontario, Canada; 3 School of Mechanical and Materials Engineering, Western University, London, Ontario, Canada; 4 Department of Diagnostic Imaging, St. Joseph’s Health Care, London, Ontario, Canada; 5 School of Health Sciences, Medical Radiation Technology Program, Fanshawe College, London, Ontario, Canada; 6 School of Health and Rehabilitation Sciences, Western University, London, Ontario, Canada; 7 Schulich School of Medicine and Dentistry, Western University, London, Ontario, Canada; IMU University, MALAYSIA

## Abstract

**Introduction/Background:**

Small amounts of malrotation can significantly decrease the diagnostic utility of a wrist radiograph. Poorly positioned wrist radiographs can result in repeat scans, additional imaging, and missed injuries. Tolerance for mistakes must be low. Radiographers, especially those with limited training, experience, or resources, may struggle to meet an acceptable standard, underscoring the need for an assistive positioning device that makes proper positioning more accessible.

**Methods:**

A human-centered design (HCD) process was used during the design of the Tru-Vu, a low-cost novel positioning aid, to assist radiographers with properly positioning of the wrist for x-rays. Initially, observations and preliminary interviews were conducted to understand the challenges faced by various end-users. The identified themes were used to guide the iterative design of the tool and training modules. To evaluate feasibility, the tool and training modules underwent testing using a cadaveric specimen.

**Results:**

Factors that affect x-ray positioning and shape device adoption were determined from the preliminary end-user interviews. Through post-intervention questionnaires, the Tru-Vu tool and training modules were found to be easy to use/understand and perceived as effective by radiographers. There was no significant difference observed in overall radiograph quality and visualization of the scapholunate (SL) interval during cadaveric feasibility testing.

**Discussion:**

This study provides insight into the HCD process used to create a novel radiographic positioning aid. The Tru-Vu tool was positively perceived by end-users; however, it did not demonstrate a significant effect on radiograph quality in cadaveric feasibility testing. Further research with live patients is required to demonstrate the clinical effectiveness of the Tru-Vu.

## Introduction

### Wrist x-ray positioning

Wrists are one of the most commonly injured anatomical regions, accounting for approximately 20% of all hospitalized fractures in Canada [1]. A wrist fracture or forced hyperextension sprain can be associated with a ruptured scapholunate (SL) ligament [2] in 32% of individuals. SL rupture, also known as SL dissociation, can lead to a form of wrist instability called SL advanced collapse (SLAC)—which is responsible for approximately 55% of wrist osteoarthritis [3]. While osteoarthritis is the most common degenerative joint disease affecting individuals over the age of 18, there are currently no effective therapeutic treatments [4]. When diagnosed early, SL ligament rupture and instability can be treated effectively through immobilization and physiotherapy or surgical intervention [5,6]. Early diagnosis is important to prevent wrist osteoarthritis [7–9].

When wrist trauma occurs or carpal instability is suspected, radiography (x-ray) is typically the first diagnostic imaging measure performed [10]. X-rays are relatively low cost, introduce minimal affiliated health risks, and are relatively accessible in lower resource areas [11]. However, x-ray sensitivity is highly dependent on anatomical positioning, including rotation, at the time of image capture [12–14]. Due to the complicated bone anatomy, this is particularly prevalent in the wrist as significant radiographic differences can be observed from small changes in wrist rotation. Koh et al. determined that a five-degree rotational margin of error exists before significant radiographic differences from the reference (i.e., perfect) lateral position can be observed [13]. Steward et al. found that 39% of lateral wrist radiographs are rejected [15,16]. Proper positioning however, is often difficult to achieve in fast paced or geographically remote clinical settings which can result in repeat x-rays, increased imaging backlog, and missed diagnoses [13]. In addition to improper patient positioning, a high rejection rate can be due to limited clinical experience during student training and a disconnect between radiographers and physicians regarding the criteria for an acceptable image [15].

Positioning aids can be used to help consistently position the wrist for standard radiographic projections. They are typically only applicable for one specific projection, cumbersome to use/store, difficult to clean, and expensive [17–19]. These limitations have hindered the wide scale adoption and usage of positioning aids in the clinical setting. More complex computer-based technologies that are currently being developed [20] are expensive and not accessible for communities with limited resources. As a result, wrist x-ray positioning is often performed using the radiographer’s best judgement.

Through a human-centered design process, the Tru-Vu wrist positioning aid and training modules were created to assist radiographers to reliably position the wrist optimally for common radiographic positions.

### Human-centered design

Human-centered design (HCD) is an iterative approach that uses collaboration to design people-centered devices, services, and systems [21]. Through methods that combine interviews and written responses with quantitative data, HCD is often implemented to solve complex health challenges in a wide range of domains such as process optimization or social innovation [21]. A unique feature of HCD is the empathy required for the various end-users throughout the interview, design, and implementation process [22,23]. Involving a wide range of end-users in the iterative design process can provide insight into various user perspectives and predictions on how individuals will interact with the solution. HCD empowers all individuals to contribute their unique perspectives and ideas, building creative confidence within the community [24]. These features of HCD can consequently result in the creation of meaningful solutions that are more likely to be accepted and implemented into practice [20,22].

IDEO, a global design company promoting community-based design and iteration, created a human-centered design field guide [22]. The field guide uses a three-step design process consisting of inspiration, ideation, and implementation stages. These stages formed the foundation for the design process in conjunction with the six ISO 9241−110 Principles of HCD which are: (1) the design is based upon an explicit understanding of users, tasks, and environments; (2) users are involved throughout design and development; (3) the design is driven and refined by user-centered evaluation; (4) the process is iterative; (5) the design addresses the whole user experience; and (6) the design team includes multidisciplinary skills and perspectives [25]. The integration of these HCD methods into the Tru-Vu design process allowed for an iterative process guided by end-user feedback.

### The development and feasibility testing of the Tru-Vu using human-centered design

The current protocols and procedures implemented in the healthcare system can be a barrier to the creation of a new device designed to disrupt them [24]. The involvement of end-users throughout the HCD process ensures solutions that work within and enhance the current environment [24], making the application of HCD principles the best route to the integration of a device into current radiography practice.

The empathetic design process used in HCD leverages diverse experiences from various individuals in the healthcare community which can inspire novel ideas to strengthen the final design [24]. As a result, a large team of end-users including radiologists, orthopedic surgeons, radiographers, and medical radiation technologists were involved throughout the duration of this study. Utilizing an HCD process, the Tru-Vu tool and training modules were developed with feedback from all end-users at each stage to improve the likelihood of integration and adoption within a clinical setting.

## Methods

The HCD process was implemented during the creation of the Tru-Vu through the three IDEO HCD stages: inspiration, ideation, and implementation [22]. Each stage consisted of varying methods (Fig 1), with consistent communication with end-users throughout the entirety of the design process.

**Fig 1:**
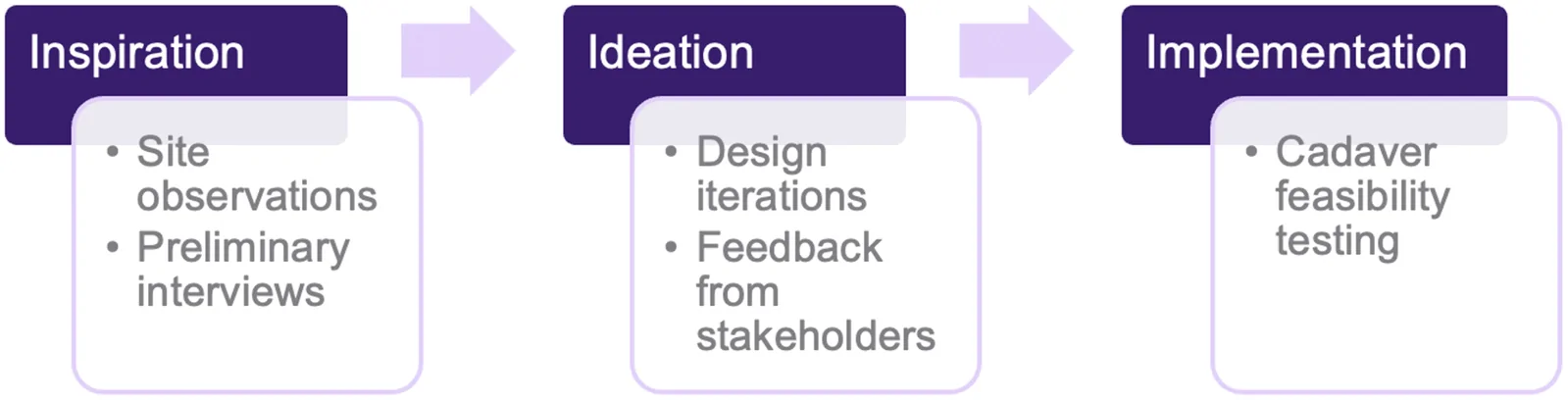
The three IDEO HCD stages with the corresponding methods performed during the design process.

### Inspiration

#### Preliminary interviews.

Healthcare workers who interact with wrist radiographs in a clinical setting were recruited between 22/04/2022 and 14/03/2024. Ethics approval for this study was obtained from the Western Health Sciences Research Ethics Board (120666). A total of three orthopedic surgeons, ten radiographers, and three radiologists were interviewed. The preliminary interviews were offered through virtual meetings, in-person meetings, and independently submitted questionnaires to maximize accessibility. The questions did not differ based on format and were specific to each group of end-users (radiographers, radiologists, and orthopedic surgeons) (S1 File). Written consent was documented and witnessed by two study team members prior to the start of each interview. Verbal consent was obtained when written consent was not possible.

The preliminary interviews were transcribed and reviewed by the primary research team. An inductive analysis was performed by two blinded research team members. Initial observations were captured through notetaking during the preliminary review of the transcripts. Keywords and phrases were tagged and grouped together to demonstrate recurring subthemes present within the data. When required, consensus between the two team members was achieved through discussion.

### Ideation

#### Synthesis of user insights into design criteria.

Themes identified from end-user interviews during the inspiration step were used to define needs and objectives for the device. Following the IDEO field guide, the themes were translated into insight statements and corresponding design criteria which provided a foundation for the rapid iterative prototyping of a human-centered device [22].

#### Feedback sessions.

Throughout the design ideation stage, multiple feedback sessions were held with a team of end-users—a radiologist, a radiographer, and two medical radiation technology educators. Meetings were held on a continual basis throughout the design process, during which collaborators provided design feedback and education regarding standard clinical wrist radiographic procedures and practices. The feedback and corresponding device iterations were documented in a device development log.

### Implementation

#### Cadaveric feasibility study.

Cadaveric feasibility testing compared the wrist positioning performed by novice and expert radiographers with and without the Tru-Vu tool and training modules. The primary outcome analyzed was the quality of PA wrist radiographs, measuring overall radiograph quality and the presence or orientation of multiple anatomical landmarks. Throughout this study, the terms radiograph quality and diagnostic quality were used interchangeably and defined as the radiograph’s utility in establishing or excluding clinical diagnoses, rather than its technical image quality. Secondary outcomes included end-user perceptions of tool usability and acceptability, measured through questionnaires, with feedback collected. Ethical approval for this feasibility study was obtained from the Western Health Sciences Research Ethics Board (123906).

Expert radiographers were defined as those with over five years of clinical practice in hospital medical imaging while novice radiographers had less than two years of experience. Three expert and three novice radiographers were recruited (n = 6) between 19/04/2024 and 14/11/2024. The study was conducted using a fresh-frozen 69-year-old male cadaveric wrist (below elbow) thawed at room temperature. The specimen was skeletally mature, with no radiographic signs of musculoskeletal disease or prior wrist trauma.

The feasibility testing followed three main stages: pre-intervention, training intervention, and tool intervention (Fig 2). A 15-minute pre-test questionnaire was used to determine baseline confidence in wrist radiography (S2 File). Following the questionnaire, participants were directed to position the cadaveric specimen for four standard radiographs: posteroanterior (PA), 45° internal oblique, 60° external oblique, and lateral. Participants then repeated the positioning procedure after observing a series of educational videos. Lastly, the Tru-Vu positioning aid was provided with tool-specific instructional videos. Time was allocated for radiographer examination of the tool and answering their questions. Post-training and tool questionnaires were administered afterwards to gain feedback and measure overall impressions of the Tru-Vu tool and training (S2 File).

**Fig 2:**
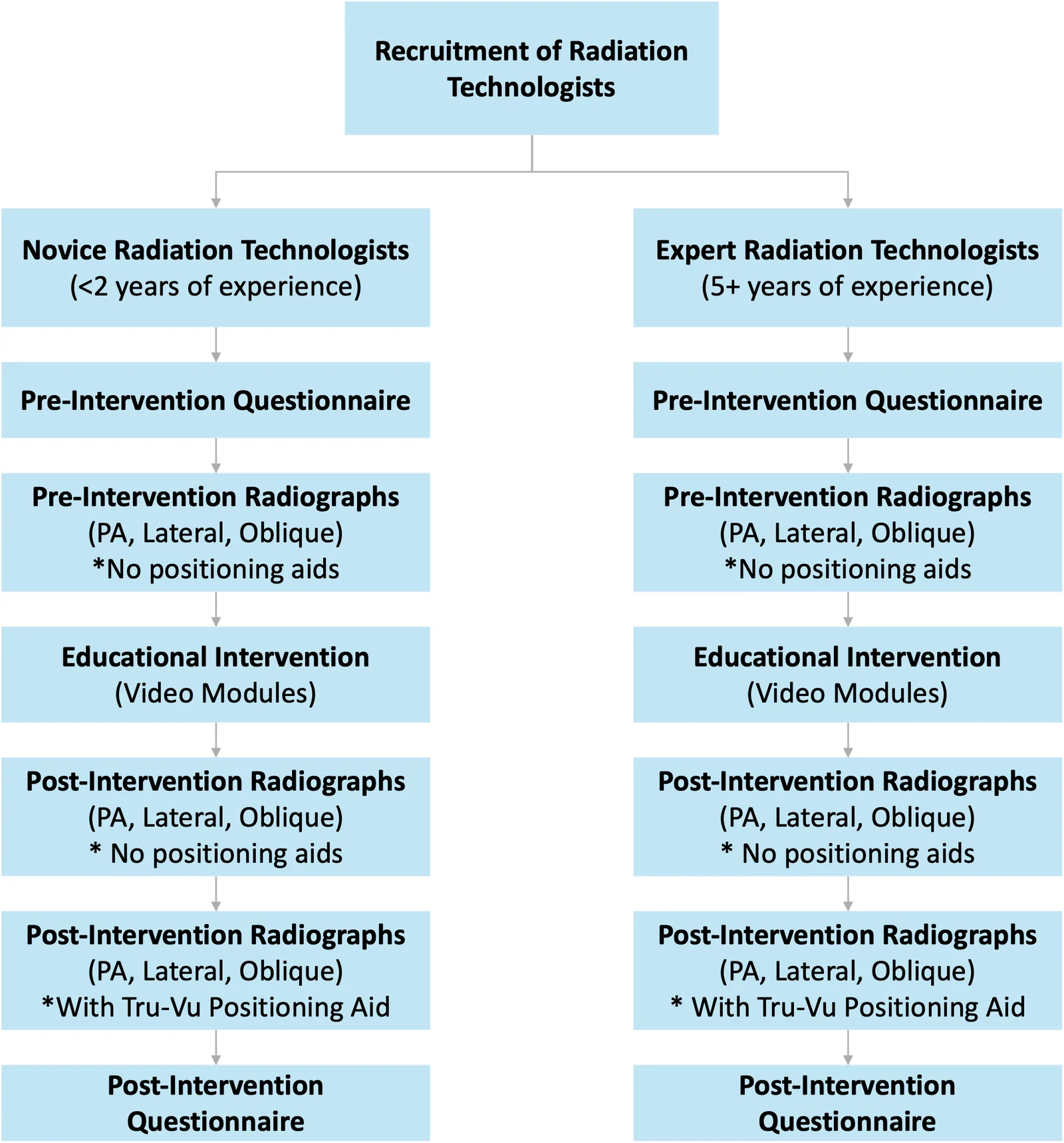
Flow diagram of the cadaveric feasibility study methodology.

Radiographs were analyzed by a blinded radiologist using the Tru-Vu Wrist Radiograph Grading Scale (S1 Fig). Previous studies have investigated the extent to which deviations from a neutral wrist position, including rotation, flexion, and extension, compromise the diagnostic quality of a radiograph [13,14]. In particular, Campbell et al. validated anatomical landmarks on PA wrist radiographs to identify malrotation [26]. These landmarks served as the foundation for the development of the Tru-Vu Wrist Radiograph Grading Scale, designed to assess the quality of standard PA wrist radiographs, especially regarding wrist malrotation. The grading criteria include 1) overall diagnostic quality, 2) open SL interval, 3) alignment of the second metacarpal with the long axis of the radius, 4) open carpometacarpal joint spaces, 5) teardrop sign, 6) balanced metacarpal heads, 7) pisiform position, and 8) pisiform overlap.

A mixed-design analysis of variance (ANOVA) using SPSS Statistics (Version 30.0) was performed to determine the relationship between radiographer experience and diagnostic quality using the Tru-Vu. Radiographer experience (novice vs expert) was treated as a between-subjects factor, and intervention (baseline, training, training and tool) as a within-subjects factor. Greenhouse-Geisser corrections were applied where sphericity was violated. Comparisons were adjusted using a Bonferroni correction and effect sizes were reported for significant effects. A confidence interval of 95% was used with an alpha level of 0.05.

Questionnaires were used to obtain quantitative evaluations of design features exhibited by the training modules and positioning tool, alongside quantitative usability feedback elicited through open-ended questions. The questions provided insight into radiographers’ experiences beyond the numerical ratings alone. Open-ended responses were reviewed and organized into common themes by the research team.

## Results

Throughout each stage of the design process—inspiration, ideation, and implementation—the device underwent multiple iterations, guided by end-user feedback.

### Inspiration

Preliminary interviews revealed overarching themes relating to the quality of wrist radiographs and factors that influence the adoption of novel positioning technologies. Six primary factors affecting radiograph quality were articulated during interviews: radiographer experience, practice setting (rural/small-town versus urban hospital centers), differences between educational training and clinical practice, the complexity of carpal anatomy, understanding of diagnostic objectives, and challenges related to patient positioning. Factors affecting technology adoption articulated included current use of positioning aids, willingness to adopt new technologies, and preferred design features of a novel positioning device.

#### Factors identified as affecting wrist radiograph quality.

1) Radiographer experience and familiarity with wrist x-ray techniques

Radiographer experience was expressed to be a key factor that can influence diagnostic quality of a radiograph. When asked *“Would you say that you are more comfortable now obtaining ‘good’ x-rays than when you first started?”*, all radiographers independently agreed. Several radiographers emphasized the importance of repeated practice to achieve mastery, while others highlighted the advantage of learning *“tips and tricks”* over time. The role of experience was further explored through the question, *“Do you think that frequency of ‘type of x-ray’ plays a role in your ability to ‘get a good x-ray?’”* Radiographers unanimously agreed that experience with a specific examination plays an important role in image acquisition.

As one radiographer explained, *“It is definitely a factor. I have loads of confidence doing standing knee x-rays, because our site does them 30 times a day for our ortho-surgeons. I have much less confidence in myself doing TMJ x-rays because they come along 3 times a year. I will still attempt a good x-ray, but my problem-solving skills… go down significantly due to lack of experience.”*

According to radiographers, both general positioning experience and examination-specific exposure contribute to a radiographer’s ability to capture a high-quality radiograph.

2) Challenges in rural and small-town setting

Responses to the question, *“As a percentage, how often would you say you see a ‘poor’ wrist x-ray?”*, suggested that the perceived diagnostic quality of wrist radiographs varies by practice setting*.* Surgeons and radiologists described observing poor-quality wrist radiographs from small-town community-based clinics approximately 40–50% of the time, with one participant estimating this proportion to be as high as 90%. This is compared to radiographs at specialized clinics or large urban hospitals, where only 10–20% were perceived to demonstrate poor diagnostic quality. Radiologists attributed this discrepancy, in part, to the differences in volume and the diversity of cases experienced. Similar to the theme of radiographer experience, participants suggested that limited exposure may restrict the development and application of advanced positioning techniques and troubleshooting strategies commonly practiced in high-volume specialized centers when faced with challenging cases.

In the words of one radiographer, *“Poor images where tech note says images are the best possible due to patient issues are almost unheard of at* [specialized hand and upper limb clinic] *but are common in clinics and community hospitals.”*

These findings suggest a disparity between the perceived diagnostic quality of radiographs performed at specialized clinics and large urban hospitals compared to rural and small-town community-based practice settings, with participants largely attributing these differences to differences in clinical exposure and experience.

3) Educational training versus clinical practice when obtaining radiographs of a complex wrist

There was consensus found among respondents that there is an insufficient amount of joint specific training provided to radiographers. When asked, *“How does your education surrounding wrist x-rays differ from actual clinical practice? Did you learn any tips?”*, radiographers reported differences between wrist positioning methods learned in their educational training versus the standard methods they currently perform in practice. One radiographer stated, “*My education differs from actual clinical practice due to the fact that we have* [a specialized hand and upper limb center] *at my clinical site which does certain views differently from how I was taught in school.”*

All radiographers except one reported learning inaccurate or inefficient positioning techniques during their training, which were then corrected during their clinical experience. This was highlighted, for example, by one radiographer*, “Originally,* [I was] *taught to put carpals flat and sort of curl up the metacarpals and fingers, I now know this is incorrect.”* Overall, participants perceived a gap between educational instruction and clinical practice specifically for wrist positioning methods.

4) Knowledge of anatomical landmarks of the wrist

Radiographers reported challenges of identifying anatomical landmarks at the wrist. When asked *“What do you think is the single greatest challenge when it comes to wrist radiography?”*, one participant highlighted the complex anatomy of the wrist: *“Correct positioning* [of] *wrist anatomy is more complex than other joints,* [as the] *keystone shape of carpus complicates alignment in x-ray.”*

To offset this difficulty, radiographers often turn to anatomical landmarks to assist them during the positioning procedure. This common practice was reinforced during the interviews with the radiographers: *“I think it is very important to use landmarks on hand and wrist* [x-rays] *to obtain proper positioning for example elevating the ulnar aspect to open joint spaces around lunate. They are not difficult to detect… every technologist has a different way to position.”* Radiographers articulated that, due to the complex carpal anatomy, their lack of familiarity with wrist landmarks during the positioning procedure can lead to malpositioning and consequently influence the diagnostic quality of a radiograph.

5) Diagnostic goals in radiographer training

In addition to the complex anatomy, surgeons and radiologists voiced a disconnect between the wrist training provided to radiographers and the diagnostic goals of physicians. When asked the question, *“What do you think is the single greatest challenge when it comes to wrist radiography?”*, one radiologist said it was making sure radiographers “*know… what radiologists/orthopaedic surgeons are doing with the radiographs,* [such that they] *have an appreciation for how the radiographs guide decision making ….”*

Physicians indicated that since radiographers are not trained in diagnosing wrist injuries, they do not know what to target during imaging, resulting in the capture of radiographs with poor diagnostic quality.

6) Factors preventing proper patient positioning

The interviews also revealed a theme of patient dependent positioning difficulties during wrist x-rays. When end-users were asked “*How would you rank the following in terms of factors prohibiting proper positioning of the wrist? 1) patient wrist ROM [range of motion] 2) available time 3) other patient related factors”* the answers varied between groups (surgeons, radiologists, and radiographers). With the exception of one radiographer, they all reported encountering challenges in positioning due to issues regarding patient understanding and awareness, as well as barriers to achieving optimal positioning in immobilized and casted patients. In addition, a radiographer acknowledged experiencing positioning challenges in suspected fracture cases due to limited mobility. All the radiographers interviewed considered available time to be the least prohibiting factor of proper wrist positioning.

#### Drivers shaping adoption of novel positioning technology.

1) Utilization of current positioning devices in wrist imaging

Currently, a wide range of positioning devices including sponges and pillows of different shapes and sizes, are used as assistive devices for wrist x-rays. When radiographers were asked the question, *“What role would you say sponges/positioning devices plays in your job?”*, respondents reported a high importance of positioning aids in clinical settings. Positioning sponges were viewed as aids improving *“image consistency and repeatability”* as well as *“to ensure anatomy is immobilized”* especially when patients are immobilized with casts or splints: *“Sponges are a huge part of my day-to-day. This is different than how I was trained, but sponges help with image consistency and repeatability.”*

However, sponges are not available at some institutions. This was demonstrated by one radiographer who reported that they do not use sponges due to a lack of access. Overall, however, the use of sponges by radiographer was reported to be high.

2) Willingness to adopt a new technology

To gain an understanding of their willingness to adopt a novel positioning aid, during the preliminary interviews radiographers were asked, *“If there was a device that helped you position the wrist, as a guide, would you use it?”.* All radiographers expressed interest in this idea, and some emphasized the importance of standardizing proper positioning to limit the occurrence of malrotation: *“Yes definitely* … *Anything that leads to consistency or standardizes best practices would be great.”*

3) Desired design features

End-users were asked how they would interact with the device in different capacities in order to understand ideal design features of a novel positioning device from various perspectives. In response to the question, *“What would you like to see in terms of innovation and device design to address this problem?”*, various desired features such as lightweight, inexpensive, portable, simple to manufacture, and sterilizable were expressed. The most common identified feature, however, was user friendliness. End-users mentioned that the device *“needs to have good instructions”*, be *“simple”*, and *“easy to use”*. Using the identified criteria, design objectives were created for the ideation stage.

In conclusion, two overarching themes emerged from the interviews: 1) Factors that affect diagnostic image quality and 2) Drivers of device adoption. The themes and their associated subthemes provided the framework for the ideation process during design of a novel positioning technology.

### Ideation

During the ideation stage, the Tru-Vu positioning aid and accompanying training modules were created to standardize wrist positioning procedures, with the goal of reducing repeat image acquisitions and facilitating the consistent capture of high-quality radiographs. The Tru-Vu tool is a wrist positioning device that provides reference surfaces (i.e., markers to indicate lateral and various degree oblique positions) for radiographers to use during the positioning procedure. The complimentary training modules provide foundational and specialized positioning information to address knowledge gaps where applicable.

#### Synthesis of user insights into design criteria.

The themes identified from the end-user interviews served as the foundation for the ideation process. Insight statements were derived from the theme of *Factors identified as affecting wrist radiograph quality* and translated into specific design criteria. The corresponding design features are outlined in Table 1. In contrast, *Drivers shaping adoption of novel positioning technology* informed the development of objectives and constraints, drawing on key phrases from the subtheme regarding desired design features (Table 2).

**Table 1:** Outline of insight statements and design features created in response to the factors affecting wrist radiograph quality identified during preliminary interviews.

Factor affecting wrist radiograph quality	Insight statement	Corresponding design feature
Radiographers’ experience and familiarity with wrist x-ray techniques	When wrist x-rays are not commonly performed, radiographers feel less confident in their ability to capture a high-quality wrist radiograph.	Positioning aid standardizes the positioning procedure for standard wrist radiographs.
Perceived challenges in rural and small-town settings	Radiographers at rural or small health centers are potentially less familiar with specialized methods for positioning the wrist.	Positioning aid makes it easy for radiographers to position the wrist with optimal rotation as performed at a specialized hand and upper limb clinic or large urban hospital.
Differences between training and practice for the complex wrist	Positioning techniques taught in school do not translate directly to a clinical environment.	Training modules highlight standard positioning methods currently employed in practice.
The understanding of anatomical landmarks of the wrist	Correctly using wrist landmarks during the positioning process can improve the quality of radiograph.	Training modules review anatomical terminology of the wrist in addition to common landmarks that can assist positioning.
Importance of the role of diagnostic goals in radiographer training	Understanding diagnostic goals can guide radiographer decision making.	Training modules discuss diagnostic goals for common wrist positions.
Prohibiting factors to proper patient positioning	A large perceived impediment to ideal positioning is limited patient mobility.	Positioning aid is complimentary to current positioning sponges used for additional wrist support when required.

**Table 2:** Preliminary list of objectives and constraints for the positioning tool.

Objectives	Constraints
• Lightweight	• Must be less than 0.5 kg
• Inexpensive	• Must cost less than $10 CAD in materials to manufacture
• Portable	• Must be less than 220 x 220 x 300 mm
• User friendly	• Must have easy to follow infographics incorporated into design
• Easy to repair	• Minimize the number of moving parts (i.e., Less than 5) • Must incorporate DFA guidelines into project
• Easy to manufacture	• 3D printable (print-in-place)
• Sterilizable	
• Adjustable to individual patient anatomy	

#### Feedback sessions.

The tool underwent seven iterations, guided by end-user feedback from the device development log (Table 3) to satisfy the desired objectives.

**Table 3:** End-user concerns with corresponding tool design iterations.

End-user concerns	End-user feedback	How concerns were addressed
Multi-use	*“The tool would be more useful if I could use it for all standard wrist x-rays, not just the PA.”*	Modified the design to include reference surfaces for lateral and oblique projections.
Design of the arm	*“The Tru-Vu arm doesn’t let me know when it is at 90°, how do I know I’m not at a slightly acute angle?”*	Restricted the arm to a 90° position along the transverse plane.
	*“The arm slides too easily; I want to be able to remove the tool from the patient and it stay in the same position in case I need to reposition the patient.”*	Altered the print orientation to increase the friction experienced during vertical translation of the arm.
Size	*“The tool is much too large to conveniently fit into my workflow.”*	Reduced the overall size of the device to occupy a 150 mm x 150 mm x 100 mm volume.

The final iteration of the tool prior to feasibility testing (Fig 3) consists of a modular arm that positions the carpal plane parallel to the imaging detector to achieve a PA projection, along with reference surfaces for other common wrist projections. The modular design of the tool increases the tool’s functionality and improves the positioning consistency for various common wrist x-rays.

**Fig 3:**
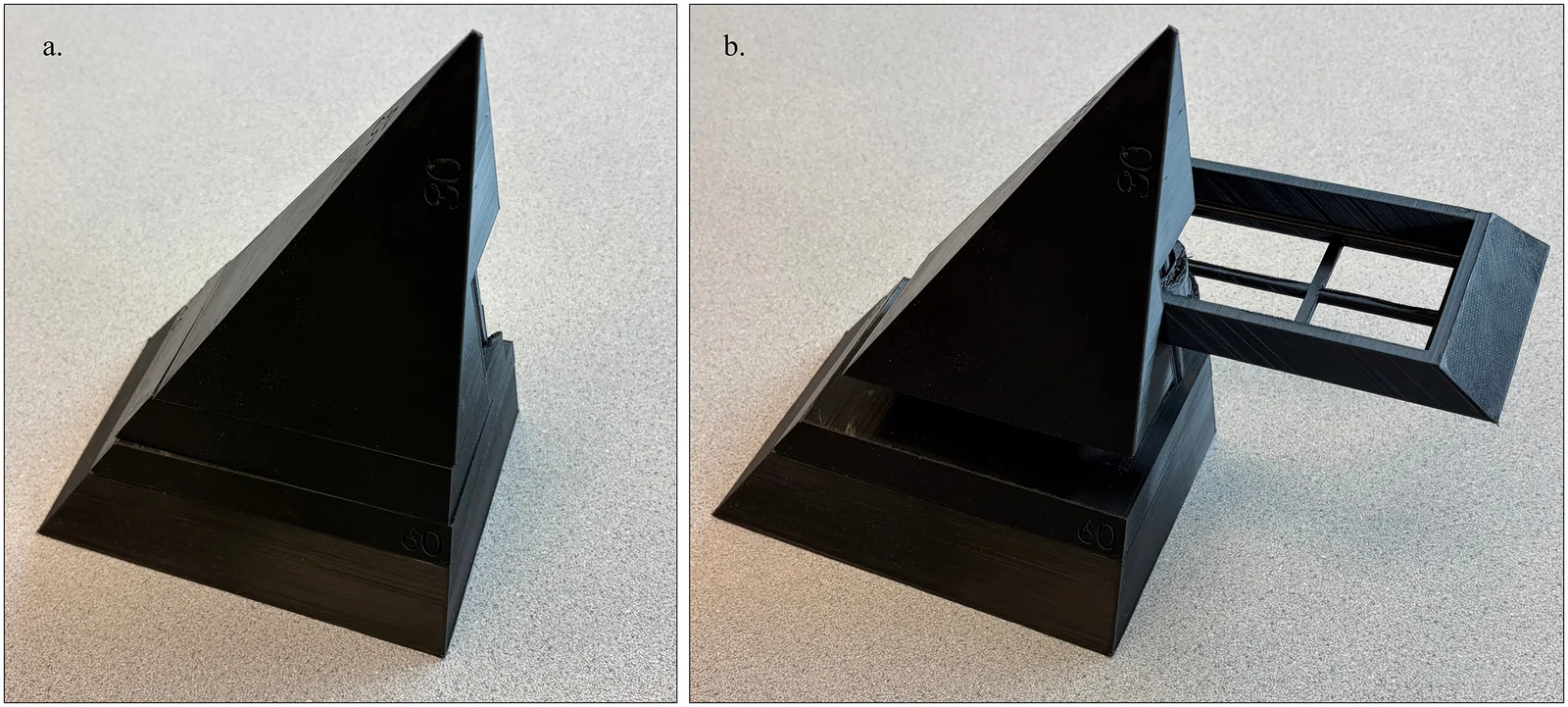
The final iteration of the Tru-Vu tool prior to feasibility testing. (a) The tool is demonstrated in the collapsed position, and (b) the arm is extended for use during PA positioning.

Similarly, the design process of the training modules consisted of three main iterations. To ensure optimal knowledge translation, end-users provided feedback regarding the terminology used, pace of content, and use of visual aids (Table 4).

**Table 4:** End-user concerns and subsequent training module design iterations.

End-user concerns	End-user feedback	How concerns were addressed
Terminology	*“I don’t know what a metacarpal body is. Radiologists don’t use that term. Do you mean the diaphysis?”*	Updated language to reflect the clinical terminology used in practice by radiographers and radiologists.
Tempo of content	*“I am wondering if these videos move a bit too quickly. I would generally slow them down a touch. Brevity is important but some parts … could use a bit more lengthy explanation”*	Slowed the pace of the videos and added an additional chapter (Chapter 0) to review foundational information on carpal anatomy and common positioning terminology.
Visual aids	*“In video 3 verbal explanations of positioning may not be understood and remembered until demonstrated with live volunteers.”*	Updated chapter 3 to include close-up images depicting the tool being used with a human wrist in the different positions.

The final design of the Tru-Vu training modules is fourteen short videos (Fig 4) categorized into five chapters. Together, the videos discuss a wide range of topics including wrist anatomy, positioning background information, x-ray principles, wrist x-ray positioning instructions, features of the Tru-Vu tool, and how to perform common wrist projections with the tool. Each module is approximately five minutes in length and contains visuals such as diagrams, animations, radiographs, and demonstration videos to assist with knowledge translation. The training modules aim to provide comprehensible specialized wrist positioning training for radiographers from all backgrounds, both with and without the use of the Tru-Vu positioning aid.

**Fig 4:**
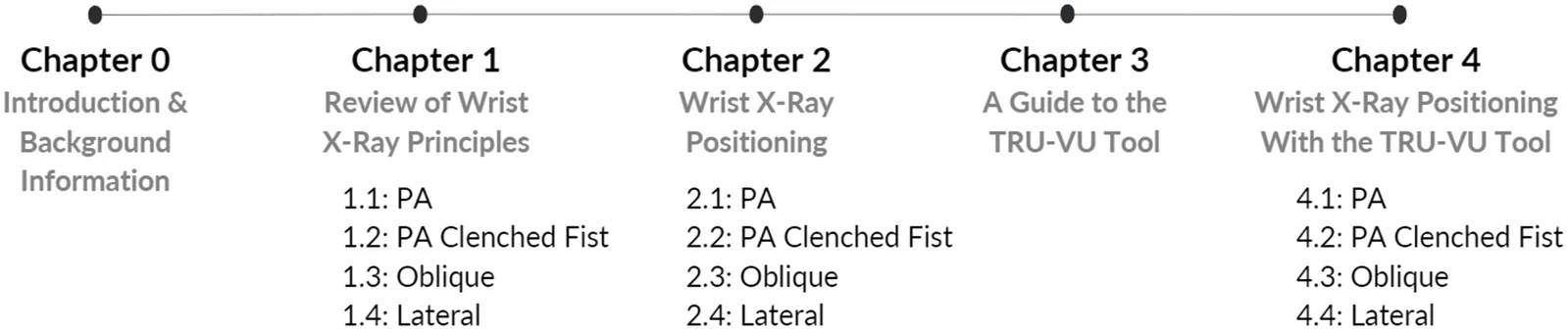
Flow diagram of the final Tru-Vu training modules.

### Implementation

#### Cadaveric feasibility study.

Separate mixed-design ANOVAs revealed no significant relationship between radiographer experience and intervention on overall diagnostic quality and SL interval scores. In addition, no significant main effects were observed between experience level (expert and novice) and intervention (baseline, training, and training and tool) (p > 0.05) (Figs 5 and 6). Additional mixed ANOVAs for the remaining graded PA landmarks demonstrated similar results, with no significant interactions or main effects (S2–S7 Figs). The post-tool intervention questionnaire provided supplementary insights from participants regarding their perception of tool usability and acceptability.

**Fig 5:**
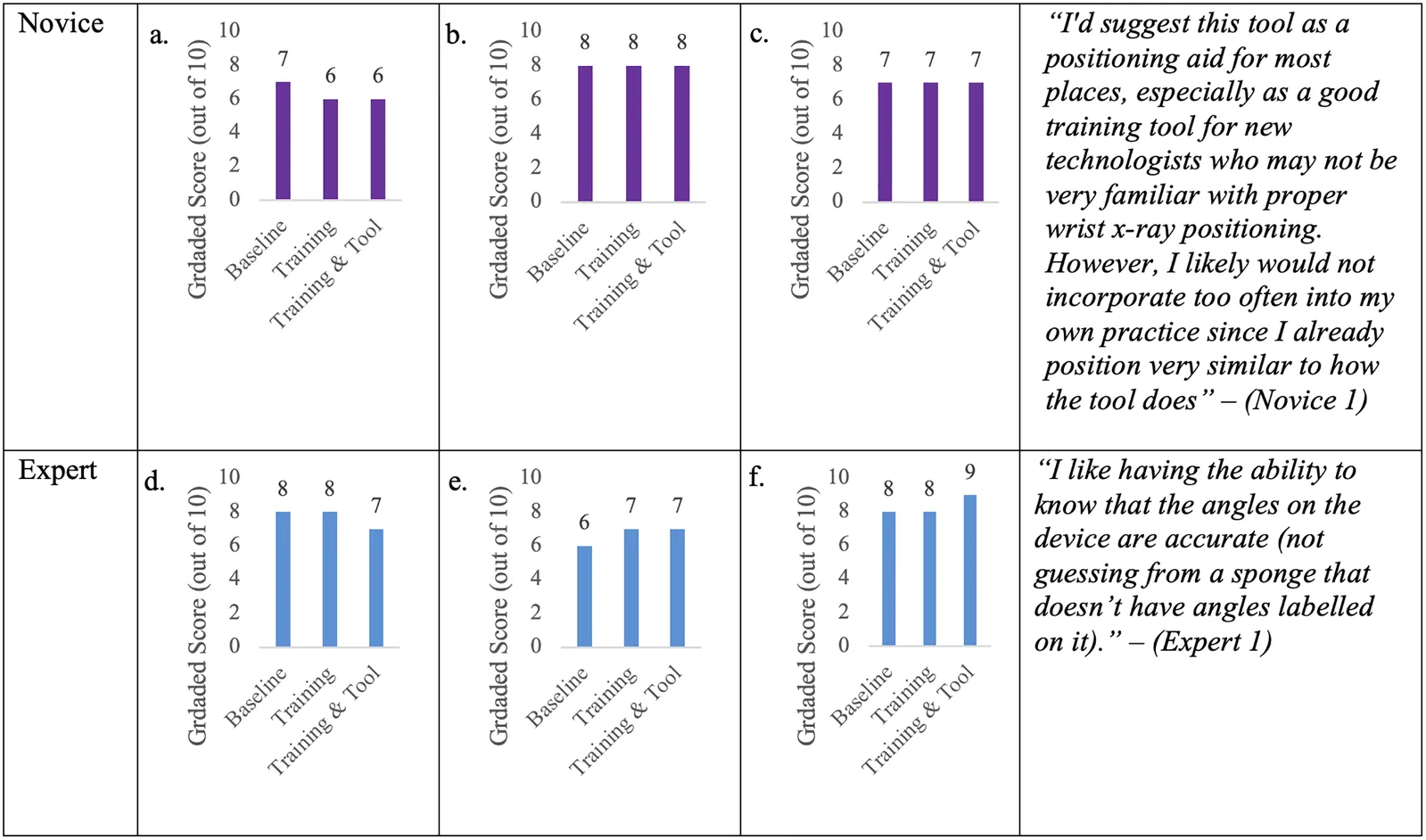
Overall diagnostic values of the radiographs captured at the different interventions by (a)-(c) novice and (d)-(f) expert participants with statements from the post-tool intervention questionnaire regarding the perceived usefulness of the device.

**Fig 6:**
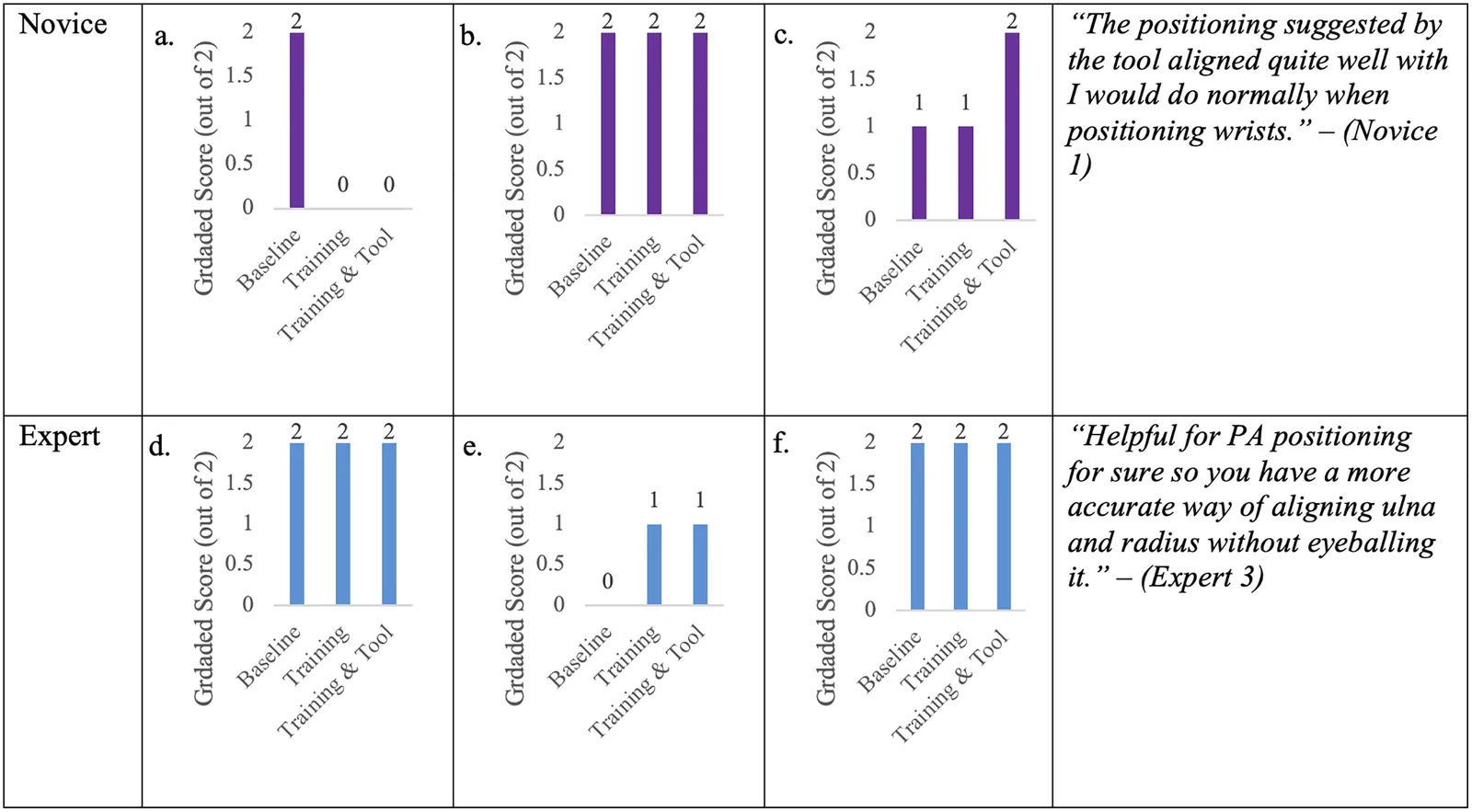
SL interval scores of the radiographs captured at the different interventions by (a)-(c) novice and (d)-(f) expert with statements from the post-tool intervention questionnaire regarding the perceived usefulness of the device to achieve an open SL interval.

Radiographers found the tool to have high effectiveness and good design features (e.g., easy to use, intuitive, and portable). In addition, they reported high likelihood of future use (Fig 7a). The training modules were thought to be an effective means of delivering positioning education to radiographers of all levels of experience (Fig 7b).

**Fig 7:**
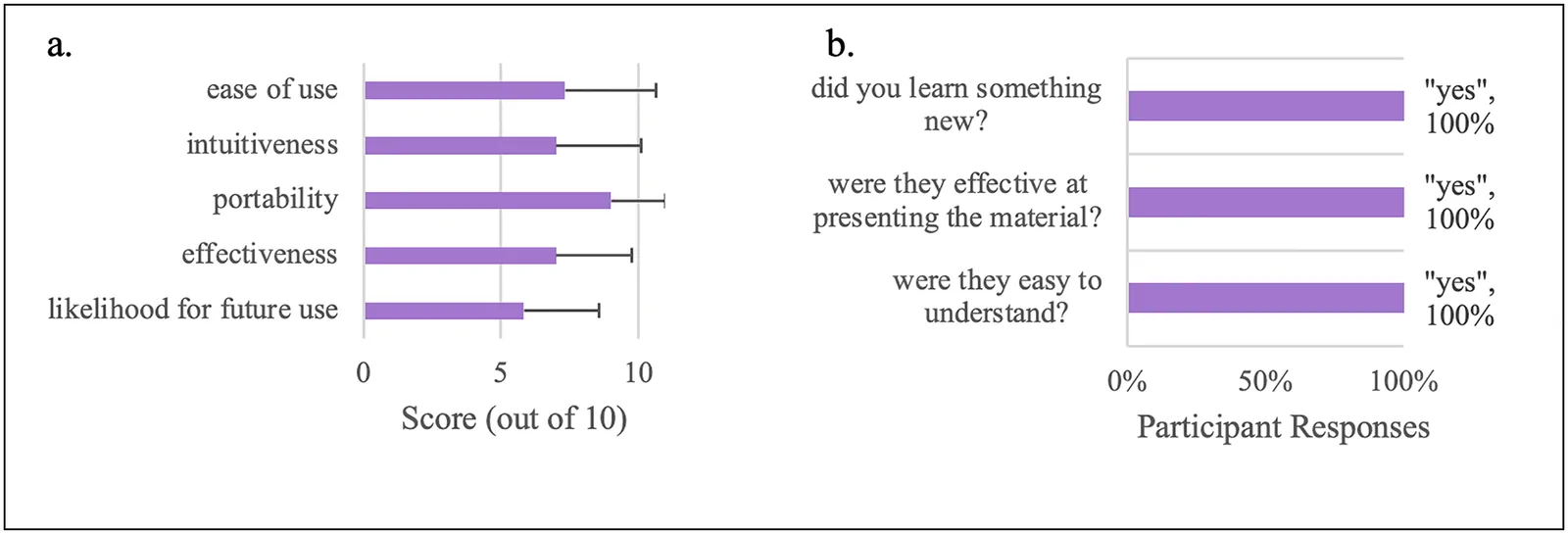
Quantitative usability data results from post-intervention questionnaires. (a) Average scores of design objectives determined through the post-tool questionnaire. (b) Responses regarding the material and design of the training modules from the post-training questionnaire.

In addition to supplementing the quantitative design feature ratings, positive and constructive feedback regarding design characteristics through the post-intervention questionnaires guided subsequent design iterations (Tables 5 and 6).

**Table 5:** Synthesized feedback from post-tool intervention questionnaire on tool design characteristics and the resulting design iterations.

Design characteristic	Feedback	Design iterations of the Tru-Vu tool
Labels indicating the oblique angles on each surface	The numbers on the different angled surfaces help to understand how to properly use the device.	N/A
Size and shape	The device was very compact, portable, and had multiple easy to use features.	N/A
	The sides are narrow which may pose an issue when trying to accurately align the wrist with the rest of the hand and forearm of a patient who has a larger wrist.	The body of the tool was modified to increase the width of the sides for patients of different sizes.
	It is difficult to fit both the tool and a sponge on the x-ray detector at the same time which will be important for individuals with limited ROM.	Updated the training modules to educate individuals on how to position a patient for the PA projection with the use of the arm and another positioning aid.
Arm	The PA arm is difficult to adjust for unique patient anatomy.	Changed the direction of the 3D printed filament in the arm and body to allow for easier adjustment.
	The PA arm sometimes swings open when not in-use.	Added a design feature to hold the PA arm in place when not being used. The arm has also been updated to be removeable.
	The design of the window on the arm is not clear.	Added additional explanation of the different features of the arm and their purpose in the training modules.
Material	The device would be more useful if it was radiolucent.	In process of testing different radiolucent materials for future design iterations.
	The device has minimal surface friction and slides easily off the detector when weight is applied on an angle (e.g., When positioning for an oblique projection).	To be taken into consideration for future designs.

**Table 6:** Synthesized feedback from the post-training intervention questionnaire on the training module design characteristics and the resulting design iterations.

Design characteristic	Feedback	Design iterations of the training modules
Speed	The material was concise and clearly explained.	N/A
	Some concepts are introduced quickly and are not summarized at the end.	Added a summary of concepts at the end of the background modules (chapter 1).
Language	Clear simple language.	N/A
Content	The importance of proper positioning with the use of certain landmarks should be elaborated.	Added diagnostic goals when discussing different landmarks or positioning methods.

## Discussion

This study followed the human-centered design (HCD) process to develop the novel Tru-Vu positioning aid and training modules. Wrist radiographs have relatively high rejection rates and due to the limitations of current solutions, many radiographers rely on their best judgement to position the wrist without the use of assistive devices. Through an iterative design process with continuous feedback from end-users, the Tru-Vu tool and training modules were developed to standardize wrist positioning and as a result improve the diagnostic quality of common wrist radiographs.

The overarching themes determined through analysis of the preliminary interviews provided a foundation for the design of the tool and training modules. The themes provided a comprehensive understanding of the factors that can affect radiograph quality as well as the drivers of adoption of a novel radiographic device as perceived by radiographers, radiologists, and orthopedic surgeons. Understanding the drivers of adoption is essential as HCD frameworks advocate for the development of technology that aligns with end-users’ existing behaviours and attitudes during adoption and use [27]. Current positioning aids were found to be preferentially used when readily available and radiographers endorsed their importance in ensuring consistency across radiographs. This finding reinforced the potential utility of a new positioning aid that overcomes the limitations of current devices used.

Standardizing radiographic projections for all radiographers irrespective of geographical location, including those in medically remote areas, was a key goal in the development of the Tru-Vu. To satisfy this requirement, print-in-place 3D printing was the selected manufacturing process. 3D printing is becoming a more mainstream manufacturing methodology within the healthcare industry through its ability to provide patients with low-cost personalized care through individualized medical devices such as protheses and implants [28]. As a result, the adoption of 3D printers is increasing within both large and small hospital environments [29]. Additionally, the use of 3D printing inherently satisfied some additional design constraints including weight and cost: parts printed with polylactic acid (PLA) through fused deposition modelling are light and relatively inexpensive.

The first iteration of the tool was created for improving visualization of the SL interval on standard PA wrist radiographs. As demonstrated by Campbell et al., just ten degrees of pronation or supination from the expert neutral PA position can result in significant differences in the visualization of anatomical landmarks, such as the SL interval [26]. However, there is no standard method for achieving this expert neutral position. For example, Merrill’s Atlas of Radiographic Positioning and Procedures by Rollins et al. instructs radiographers to flex the hand to create a ten-to-fifteen-degree angle with the image receptor, placing the wrist in a neutral position [30], while Radiographic Image Analysis by Martensen instructs radiographers to place the wrist closer to the image receptor by flexing the fingers [31]. Using different methodologies, both techniques aim to ensure the radius and ulna are parallel to the image receptor to allow for clear visualization of the SL interval. The tool was designed to standardize the positioning procedure by providing a reference surface for radiographers to use when pronating the ulnar aspect of the wrist. Based on feedback from end-users, the tool was further iterated to assist with additional common wrist projections.

In addition to the overall diagnostic quality, SL interval scores were reported by end-users as a primary metric due to its high sensitivity to malrotation. Even slight rotation from the neutral PA position, whether pronation or supination, can lead to significant differences in radiographic SL interval length [26]. The teardrop sign, balanced metacarpal heads, and pisiform position landmarks however, are not as sensitive to pronation but were included on the Tru-Vu Wrist Radiograph Grading Scale to provide additional information regarding malrotation due to supination [26]. The remaining landmarks of pisiform overlap, alignment of the second metacarpal with the long axis of the radius, and open carpometacarpal joint spaces were not shown to demonstrate significant differences by Campbell et al. but are considered markers of proper PA positioning by radiographers [26].

The cadaveric testing performed during the implementation stage provided insight into the feasibility of the tool and training modules through feedback from end-users. Although no significant differences were found in the overall diagnostic quality between the different interventions, radiographer feedback provided useful insight. One common design feedback provided by radiographers was that the tool should be radiolucent. As the Tru-Vu tool is not radiolucent, after assisting with the positioning procedure it must be removed from the field of view before image capture. Improving the radiolucency of the tool would allow radiographs to be captured while the tool is present in the field of view, decreasing the need for additional positioning sponges and eliminating the risk of position deviation during removal of the tool from the field of view. To achieve tool radiolucency, different 3D printing materials and methods of manufacturing are being explored.

There were limitations to this study. As the cadaveric feasibility testing was performed at a specialized hand and upper limb clinic, both novice and expert radiographers were previously trained to incorporate expert wrist positioning techniques into their standard practice. Pronation of the ulnar aspect of the wrist, the technique employed by the tool to improve the radiographic view of the SL interval, is considered standard practice by radiographers at the specialized hand and upper limb clinic. This theme was common among radiographers: *“Most of my colleagues are very diligent about elevating the ulnar aspect of the wrist,”* and *“I didn’t personally feel that this part was necessary to help in my positioning.”* The pre-existing alignment of positioning techniques with the tool may have limited the observed impact of the tool and training and introduced bias at baseline. Additionally, radiographers rated likelihood of future use lower on average when compared to perceived effectiveness and design features including ease of use, intuitiveness, and portability. Similarly, these findings may be explained by the minimal perceived need for additional assistance to achieve high-quality radiographs due to the expert techniques regularly employed by radiographers at the specialized clinic.

Another limitation of this study was associated with the use of isolated cadaveric wrists during the feasibility testing of the Tru-Vu. Positioning an isolated cadaveric wrist is fundamentally different from positioning a live patient. Along with the inability to simulate pain and limited range of motion experienced in real patient conditions, cadaveric wrists require external positioning aids to maintain set positions. After participants performed the positioning procedure, positioning sponges were used to maintain the position of the cadaveric wrist during image capture across all interventions, including with the use of the tool. This technique was used by radiographers, often with the same sponges across interventions, which may have contributed to similar overall diagnostic quality and landmark scores.

The primary purpose of this study was to describe the HCD process used to create the Tru-Vu positioning aid and training modules through the inspiration, ideation, and implementation stages. Principles of HCD, including collaboration, iteration, and systems thinking, are highlighted through the end-user feedback that guided the design process and informed subsequent iterations of the Tru-Vu. However, due to the limitations of cadaveric specimens and high pre-study wrist-specific skill level of the radiographers used for feasibility testing, this study cannot be considered an accurate assessment of clinical utility and effectiveness. More evidence is required to determine the clinical efficacy of the Tru-Vu. Following this preliminary design study, future work should involve testing with live patients and inclusion of inexperienced and resource challenged radiographers to better understand the Tru-Vu’s application in a clinical setting.

## Conclusion

The development of the novel Tru-Vu positioning aid and training modules used a HCD approach in response to the challenge of capturing high-quality wrist radiographs. Throughout the three design stages—inspiration, ideation, and implementation—continuous collaboration with a team of radiologists, orthopedic surgeons, and radiographers provided the team with a holistic understanding of the initial challenge and the impact of different design decisions along the way. This HCD process led to the development of the current iteration of the Tru-Vu tool and training modules in which radiographers perceived to be effective in enhancing wrist-specific positioning knowledge and practice. Due to the limitations of this study, including the use of cadaveric specimens and the high wrist-specific pre-study skill level of all radiographer participants, future testing with live human subjects is planned in a variety of settings to further investigate the Tru-Vu’s ability to improve diagnostic quality in a clinical setting.

## Supporting information

S1 File Preliminary interview questionnaires. (PDF)

S2 File Cadaveric feasibility pre- and post-intervention questionnaires. (PDF)

S1 Fig Tru-Vu wrist radiography grading scale. (TIF)

S2 Fig Graded alignment of second metacarpal with radius in the radiographs captured at the different interventions by (a)-(c) novice and (d)-(f) experts. (TIF)

S3 Fig Graded open carpometacarpal joint spaces in the radiographs captured at the different interventions by (a)-(c) novice and (d)-(f) experts. (TIF)

S4 Fig Graded teardrop sign in the radiographs captured at the different interventions by (a)-(c) novice and (d)-(f) experts. (TIF)

S5 Fig Graded balanced metacarpal heads in the radiographs captured at the different interventions by (a)-(c) novice and (d)-(f) experts. (TIF)

S6 Fig Graded pisiform position in the radiographs captured at the different interventions by (a)-(c) novice and (d)-(f) experts. (TIF)

S7 Fig Graded pisiform overlap in the radiographs captured at the different interventions by (a)-(c) novice and (d)-(f) experts. (TIF)

S1 Dataset Minimal Dataset contains data from cadaveric feasibility testing, including graded radiograph scores and post-intervention questionnaire results. (PDF)
