# Supplementary material for: Standardizing wrist x-rays: The human-centered design and evaluation of a wrist x-ray positioning tool
Source: PLoS One. 2026 Oct 5;21(10):e0359437. doi: 10.1371/journal.pone.0359437 (PMC13637914; doi:10.1371/journal.pone.0359437)
Supplement: Supplementary file 1 [file pone.0359437.s001.pdf]

S1 File

Radiologist & orthopedic surgeon interview questions

1) Talk me through what you look for when you first review a wrist x-ray.
2) As a percentage, how often would you say you see a ‘poor’ wrist x-ray?
3) What do you think are some reasons that technologists might have difficulty obtaining a ‘good’ x-ray?
4) How would you rank the following in terms of factors prohibiting proper positioning of the wrist? 1) patient wrist ROM 2) available time 3) other patient related factors.
5) GE’s new slogan for x-ray imaging is, “Make your first image count?” What do you think they are referring to?
6) There are some radiographic markers that technologists use to obtain ‘true AP’s” or lateral x-rays. Do you use these markers place in ensuring proper positioning of the wrist? Do you look for these same markers?
7) What do you think the single greatest challenge is when it comes to wrist radiography?
8) Do you think that radiographic markers are sensitive to rotation? How much rotation would you say would be too much rotation?
9) Can you think of a time when you were reviewing a malpositioned wrist radiograph and you found it particularly challenging to read it. Can you think of a time when you were reviewing a malpositioned wrist radiograph and you found it particularly challenging to read it. What compromises did you make?
10) Do you think some sort of ‘indicator’ would be useful to you to indicate that some malrotation has occurred?

11) What would you like to see in terms of innovation and device design to address this problem?

Radiation technologist interview questions

1) On a scale of 0-10, how comfortable would you say you are at obtaining 'good' wrist x-rays?
2) On a scale of 1-10, (10 being extremely easy), how easy would you say it is to position the wrist correctly and obtain the corresponding image you are trying to capture?
3) What are some reasons that you might have difficulty obtaining a 'good' x-ray?
4) How would you rank the following in terms of factors prohibiting proper positioning of the wrist? 1) patient wrist ROM 2) time 3) other patient related factors.
5) There are some radiographic markers that technologists use to obtain 'true AP's" or lateral x-rays. What role do you think these markers place in ensuring proper positioning of the wrist? Are they difficult to detect?
6) What do you think the single greatest challenge is when it comes to wrist radiography?
7) Would you say that you are more comfortable now obtaining 'good' x-rays than when you first started?
8) What role would you say sponges/positioning devices plays in your job?
9) Do you think that frequency of 'type of x-ray' plays a role in your ability to 'get a good x-ray'?
10) How does your education surrounding wrist x-rays differ from actual clinical practice? Did you learn any tips?

11) Think of a recent time you took a wrist x-ray. How did it go? Talk me through your process.
12) If there was a device that helped you position the wrist, as a guide, would you use it?
13) Do you think that radiographic markers are sensitive to rotation? How much rotation would you say would be too much rotation?
14) Can you think of a time when it was particularly challenging to position the wrist or when the x-rays themselves were just not quite ‘right’. What was challenging and how did you work through this? What compromises did you make?
15) What would be some things you would like to see improved in your job? How would these innovations improve your health care delivery?
16) What would you like to see in terms of innovation and device design to address this problem?
