# Supplementary material for: Standardizing wrist x-rays: The human-centered design and evaluation of a wrist x-ray positioning tool
Source: PLoS One. 2026 Oct 5;21(10):e0359437. doi: 10.1371/journal.pone.0359437 (PMC13637914; doi:10.1371/journal.pone.0359437)
Supplement: Supplementary file 2 [file pone.0359437.s002.pdf]

S2 File

Cadaveric feasibility pre-intervention questionnaire

1) How many years of experience do you have of taking X-ray images of the hand/wrist?
2) On a scale of 0-10, how comfortable would you say you are at obtaining ‘good’ wrist X-rays?
3) On a scale of 1-10, (10 being extremely easy), how easy would you say it is to position the wrist correctly and obtain the corresponding image you are trying to capture?
4) What are some reasons that you might have difficulty obtaining a ‘good’ X-ray?
5) How would you rank the following in terms of factors prohibiting proper positioning of the wrist?
    a. Patient wrist range of motion (ROM)
    b. Time
    c. Other patient related factors (eg casting, position comprehension, etc)
6) GE’s new slogan for X-ray imaging is, “Make your first image count”. What does this mean to you?
7) There are some radiographic markers that technologists use to obtain "true AP’s" or lateral X-rays. What role do you think these markers play in ensuring proper positioning of the wrist? Are they difficult to detect?
8) What do you think the single greatest challenge is when it comes to wrist radiography?
9) Would you say that you are more comfortable now obtaining ‘good’ X-rays than when you first started?
    a. yes

    b. no
    c. other

10) What role(s) would you say sponges/positioning devices play in your job?
11) Do you think that frequency of ‘type of X-ray’ plays a role in your ability to get a 'good' X-ray?
12) How does your education surrounding wrist X-rays differ from actual clinical practice? Did you learn any tips?
13) Think of a recent time you took a wrist X-ray. How did it go? Talk me through your process….
14) If there was a device that helped you position the wrist, as a guide, would you use it?
    a. Yes
    b. No
    c. Other
15) Do you think that radiographic markers are sensitive to rotation? How much rotation would you say would be too much rotation?

16) Can you think of a time when it was particularly challenging to position the wrist or when the X-rays themselves were just not quite ‘right’. What was challenging and how did you work through this? What compromises did you make?
17) What would be some things you would like to see improved in your job? How would these innovations improve your health care delivery?

Cadaveric feasibility post-training intervention questionnaire

1) How did you find the training material?
2) Did the training module include appropriate and adequate visual aids?
3) Was it easy to understand the language in the printed documents?
4) Which format of the training material (printed poster) or online module?
5) Which training materials did you find most interesting?
6) Which elements of the content do you feel require improvement?
7) Overall, were the online lessons and effective means of presenting the material?
8) Do you think you could have just used the poster to interact with this material?
9) Did you learn anything today? Why or why not?
10) How likely, on a scale of 1-10, with 10 being very likely to recommend, would you recommend this training to a colleague?

Cadaveric feasibility post-tool intervention questionnaire

1) What was your initial reaction to the device?
2) What were some things about the device you really liked?

3) What were some things that you would like to see changed or improved about the design?
4) On a scale of 0-10, with 10 being the best, how easy would you say the tool is to use?
5) Can you comment on what made it easy or not easy to use?
6) On a scale of 0-10, with 10 being the best, how intuitive would you say the tool is to use?
7) Can you comment on what made it intuitive or not?
8) On a scale of 0-10, with 10 being the best, how portable would you say the tool is to use?
9) Can you comment on what made it portable or not?
10) On a scale of 0-10, with 10 being the best, how effective would you say the tool is to use?
11) Can you comment on what made it an effective or not effective wrist positioning tool?
12) On a scale of 0-10, with 10 being the best, how intuitive would you say the tool is to use?
13) On a scale of 0-10, with 10 being the best, how likely would you be to incorporate this tool into your practice? Why or why not?
14) If you were to incorporate, would you expect that you would use this for 0-3 months, 0-6 months, for over a year, or always?
