# Supplementary material for: Standardizing wrist x-rays: The human-centered design and evaluation of a wrist x-ray positioning tool
Source: PLoS One. 2026 Oct 5;21(10):e0359437. doi: 10.1371/journal.pone.0359437 (PMC13637914; doi:10.1371/journal.pone.0359437)
Supplement: Supplementary file 3 [file pone.0359437.s010.pdf]

<u>Cadaver Feasibility Testing Dataset</u>

Graded Radiograph Scores

The Tru-Vu Wrist Radiograph Grading Scale was used by a blinded radiographer to assess the PA radiographs captured by each participant at the different interventions.

	Participant A			Participant B			Participant C			Participant D			Participant E			Participant F		
Experience (Expert = E, Novice = N)	N			N			N			E			E			E		
Intervention (Baseline = B, Training = Tr, Tool = T)	B	Tr	B	Tr	T	T	B	Tr	T	B	Tr	T	B	Tr	T	B	Tr	T
Overall diagnostic value	7	6	7	7	7	6	8	8	8	8	8	7	6	7	7	8	8	9
1. Open scapholunate interval	2	0	1	1	2	0	2	2	2	2	2	2	0	1	1	2	2	2
2. Alignment of the second metacarpal with the long axis of the radius	0.5	0.5	0.5	0.5	0.5	0.5	0.5	0.5	0.5	0.5	0.5	0.5	0.5	0.5	0.5	0.5	1	0.5
3. Open CMC joint spaces	1	1	1	1	1	1	1	1	1	2	1	1	1	1	1	1	1	1
4. Teardrop Sign	1.5	1	1	1.5	1.5	0.5	1.5	2	2	1.5	1.5	1	1	1.5	1	1.5	2	2
5. Balanced Metacarpal Heads	0.5	0	0	0	0	0	1	1.5	1	1	1.5	1	0	0	0	1	0.5	0.5
6. Pisiform Position	0	0.5	1	0	0	0.5	0	0	0	0	0	0.5	0.5	0.5	0	0	0	0
7. Pisiform Overlap	0	0	0	0	0	0	0	0.5	0.5	0	0.5	0	0	0	0	0	0.5	1

Tool Quantitative Usability Data

The post-tool intervention questionnaire, completed by all participating radiographers, provided supplementary quantitative usability data regarding the usability and acceptability of the tool.

	Participant A	Participant B	Participant C	Participant D	Participant E	Participant F
Experience (Expert = E, Novice = N)	N	N	N	E	E	E
Ease of Use	9	8	9	7	2	9
Intuitiveness	6	8	8	8	2	10
Potability	5	10	9	10	10	10
Effectiveness	8	7	8	8	1	10
Likelihood for future use	6	3	8	7	1	10

Training Module Supplementary Data

The post-training intervention questionnaire, completed by all participating radiographers, provided supplementary data regarding the usability and acceptability of the training modules.

	Participant A	Participant B	Participant C	Participant D	Participant E	Participant F
Experience (Expert = E, Novice = N)	N	N	N	E	E	E
Did you learn something new?	YES	YES	YES	YES	YES	YES
Were they effective?	YES	YES	YES	YES	YES	YES
Were they easy to understand?	YES	YES	YES	YES	YES	YES
